# Curated benchmark dataset for ultrasound based breast lesion analysis

**DOI:** 10.1038/s41597-024-02984-z

**Published:** 2024-01-31

**Authors:** Anna Pawłowska, Anna Ćwierz-Pieńkowska, Agnieszka Domalik, Dominika Jaguś, Piotr Kasprzak, Rafał Matkowski, Łukasz Fura, Andrzej Nowicki, Norbert Żołek

**Affiliations:** 1 Institute of Fundamental Technological Research, Polish Academy of Sciences, Pawinskiego 5B, 02-106 Warsaw, Poland; 2 Maria Sklodowska-Curie National Institute of Oncology - National Research Institute Branch in Krakow ul, Garncarska 11, 31-115 Kraków, Poland; 3 Breast Unit, Lower Silesian Oncology, Pulmonology and Hematology Center, pl. Ludwika Hirszfelda 12, 53-413 Wrocław, Poland; 4 Department of Oncology, Wrocław Medical University, Wrocław, Poland

**Keywords:** Breast cancer, Breast cancer

## Abstract

A new detailed dataset of breast ultrasound scans (BrEaST) containing images of benign and malignant lesions as well as normal tissue examples, is presented. The dataset consists of 256 breast scans collected from 256 patients. Each scan was manually annotated and labeled by a radiologist experienced in breast ultrasound examination. In particular, each tumor was identified in the image using a freehand annotation and labeled according to BIRADS features and lexicon. The histopathological classification of the tumor was also provided for patients who underwent a biopsy. The BrEaST dataset is the first breast ultrasound dataset containing patient-level labels, image-level annotations, and tumor-level labels with all cases confirmed by follow-up care or core needle biopsy result. To enable research into breast disease detection, tumor segmentation and classification, the BrEaST dataset is made publicly available with the CC-BY 4.0 license.

## Background & Summary

Breast cancer is the most commonly diagnosed cancer in women worldwide accounting for over 2.2 million new cases and resulting in over 650000 deaths in 2020^1^. In breast examination, ultrasound, mammography and magnetic resonance imaging are the most prevalent imaging modalities. Among them, ultrasound examination is gaining affordability and wide availability. However, it is also a highly operator-dependent modality, and depending on the breast structure or tumor type, the difficulty of spotting critical findings is varying^2^. Therefore, a reliable breast ultrasound examination requires a radiologist experienced in breast diagnostic imaging following the BI-RADS guidelines of the American College of Radiology (ACR)^3^. Although the atlas describes the signs of different breast abnormalities, interobserver agreement and intraobserver repeatability in breast assessment have been reported as poor^4^ or at most moderate^5^. To address the issue, data-driven decision systems should be developed to support radiologists’ diagnoses.

Machine learning models have been developed for different clinical applications in breast examinations, such as automatic cancer detection^6^, segmentation^7,8^ and classification into malignant and benign breast tumors^9,10^. High-quality data is a key element for selecting features, developing theoretical models and augmented inference methods^11^. The dataset quality and reliability are particularly important in healthcare fields, where inaccuracies can lead to image misinterpretation and retard correct diagnosis^12^. Furthermore, models often underperform when they are tested on datasets collected using different devices at different sites due to domain shift^13^. It can be caused by differences in the set-up of the ultrasound machines or algorithms used for image enhancement. The process of building a dataset that satisfies the requirements is costly and time-consuming due to some constraints: (1) scans saved in the hospital’s Picture Archiving and Communication System (PACS) are non-anonymized what makes them hard to access; (2) manual annotation by an experienced radiologist is expensive; and (3) there is no efficient system for storing, labeling and annotating medical image sets.

Six breast ultrasound datasets such as Open Access Series of Breast Ultrasonic Data^14^, Breast Ultrasound Lesions Dataset^15^, Medical Image Database^16^, Breast Ultrasound Videos^17^, Breast Ultrasound Dataset^18^, and Breast Ultrasound Images Database^19^ have been published in recent years. One of them^17^ consists of video frames with rectangular bounding boxes, the lack of manual annotations excluded it from further consideration. The largest one^18^ contains 780 images, but unfortunately, more than 40% have significant defects: duplicated images, sometimes classified differently, axilla images instead of breast images, presence of measurement markers or Color Doppler region of interest in the image, etc.^20^. All other datasets are smaller in size but they also contain images limiting their utility. Of the five datasets, only one includes annotations of multiple tumors in the image and can be used for the detection and segmentation of abnormalities. Furthermore, only two datasets were labeled with a diagnosis (with three diagnoses^15^ and with eight diagnoses^16^), while none of them associate a diagnosis with labels of critical findings. A summary of the published datasets and the dataset^21^ presented here is provided in Table 1. As already published datasets are not detailed enough and a benchmark reliable dataset has not yet been published, the field of breast ultrasound datasets remains unexploited.

**Table 1:** An overview of publicly available breast ultrasound datasets and the dataset presented in this work.

	Open Access Database of Raw US Signals^14^ (last acc. May 31 2023)	Medical Image Database^15^ (last acc. May 31 2023)	Breast Ultrasound Lesions Dataset^16^ (last acc. May 31 2023)	Breast Ultrasound Dataset^18^ (last acc. May 31 2023)	Breast Ultrasound Images Database^19^ (last acc. Aug 31 2023)	presented dataset (BrEaST)
Release year	2017	2018	2018	2020	2023	2023
Datatype	postbeamformed RF signals	images	images	images	images	images
No. of cases (benign/malignant/normal)	100 (48/52/0)	180 (120/60/0)	163 (110/53/0)	780 (487/210/133)	232 (109/123/0)	256 (154/98/4)
No. of ultrasound devices	1	1	1	2	1	4
No. of radiologists	1	3	not stated	not stated	1	4
Histopathological confirmation	yes	not stated	yes	not stated	partially (cytology included)	yes
Diagnosis	not stated	60 cancers, 60 cysts, 60 fibroadenomas	65 cysts, 39 fibroadenomas, 40 invasive ductal carcinomas, 4 ductal carcinomas in situ, 2 invasive lobular carcinomas, 2 papilomas, 3 lymph nodes, 1 lymphomas, 7 unknown	not stated	no	33 histological diagnoses (details in Table 4)
Physical size of image	possible to compute	no	no	no	no	yes (pixel size)
Segmentation of multi-lesion images	no	yes	no	no	no	yes
BI-RADS category	yes	no	no	no	no	yes
BI-RADS features	no	no	no	no	no	yes
License	non-commercial research; cite the source	cite the source	non-commercial research; sign release agreement via email; cite the source	CC0: Public Domain; cite the source	cite the source	Attribution 4.0 International (CC-BY 4.0)
Dataset DOI/version control	no	no	no	no	no	yes
Remarks (examples of issues)	• duplicated image (30NH first scan plane = 31NH second scan plane) • no annotation of multi-lesion images (e.g. 201AT both planes)	• duplicated image (e.g. Case-177 = Case-210) • missing tumor annotation (e.g. Case-1) • measurements markers (e.g. Case-1) • missing B-mode image (e.g. Case-170) • visible biopsy needle (e.g. Case-227)	• no annotation of multi-lesion images (e.g. 78)	Detailed issues are listed in^20^ • duplicated images (e.g. normal 39 = normal 48 = normal 56) • no annotation of multi-lesion images (e.g. benign 387) • axilla images (e.g. benign 306) • annotations in images (e.g. normal 121) • measurements markers (e.g. benign 433) • Color Doppler region of interest (e.g. benign 277)	• fine needle aspiration cytology used as a histopathological confirmation method • no skin layer - cropped images (e.g. malignant 16) • tumor exceeding image size (e.g. benign 66) • measurements markers (e.g. benign 27) • visible biopsy needle (e.g. malignant 122)	

In this paper, we present an expert-annotated dataset^21^ of 256 ultrasound images of the breast. The dataset consists of images of 154 benign tumors, 98 malignancies and 4 normal breasts. To provide generality to the dataset, images were collected by five radiologists at medical centers in Poland in 2019–2022. All images were manually annotated and labeled by radiologists via a purpose-built cloud-based system. The dataset contains patient-level labels, image-level annotations, and tumor-level labels with all tumors confirmed by follow-up care or biopsy result. In particular, the first stage of data collection considered clinical data as patient-level labels, i.e. age, breast tissue composition, signs and symptoms. The second part was adding image-level freehand annotation identifying the tumor and other abnormal areas in the image. Then, the tumor and image were labeled with BIRADS category, BIRADS descriptors, and interpretation of critical findings as presence of breast diseases. The final labels regarded the method of verification, tumor classification and histopathological diagnosis (33 diagnoses). Compared to the publicly available datasets (Table 1), the BrEaST dataset includes annotations of multi-lesion images, core needle biopsy results and is labeled for BIRADS features to support the BIRADS category.

## Methods

**Fig. 1:**
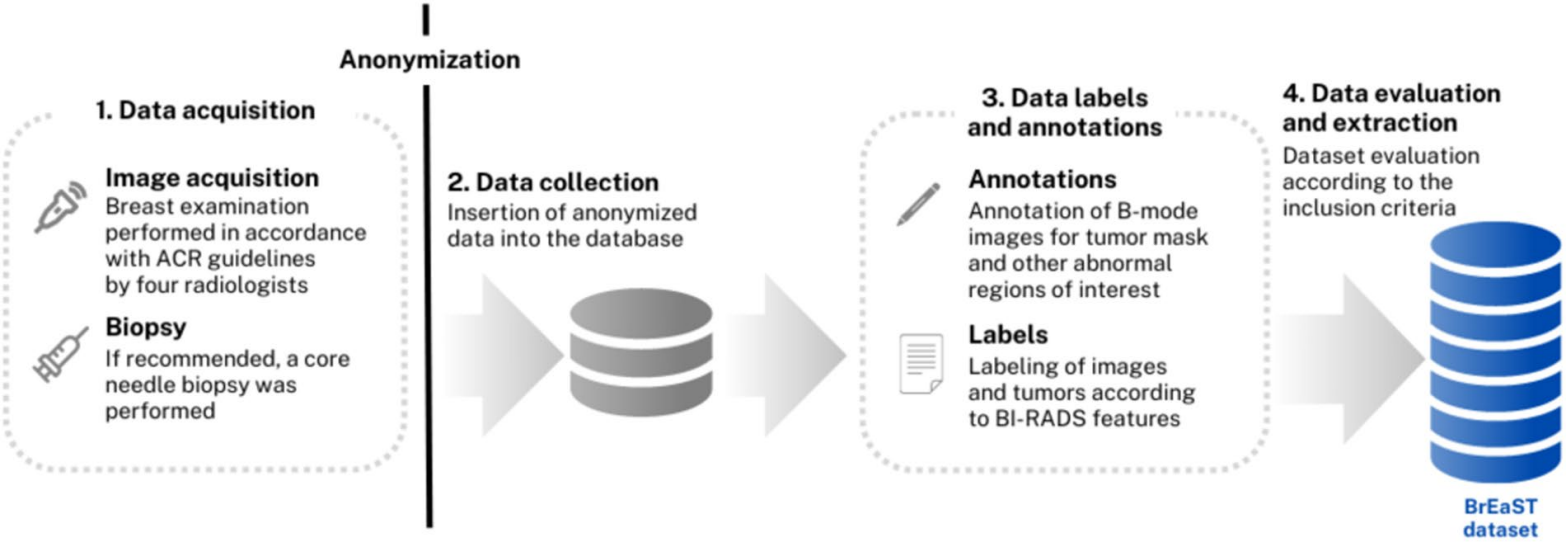
Overview of the dataset workflow: (1) images and clinical data acquisition at medical centers; (2) anonymization, transfer and insertion into the database; then (3) preparation of manual (freehand) annotations and labels; finally, (4) evaluation and export as the BrEaST dataset.

The breast ultrasound images from 256 patients were collected at medical centers in Poland in 2019–2022. Ethical approval for this study was obtained from the Bioethics Committee at the Lower Silesian Chamber of Medicine no. 2/BNR/2022. The requirement of obtaining written informed consent from patients was forgone because retrospective data collection has not impacted the standard diagnostic procedures and all data has been anonymized before being entered into the database. The data transfer, annotation and labeling were conducted via a purpose-built web-based system at the Institute of Fundamental Technological Research of the Polish Academy of Sciences in Poland. The scheme of workflow is shown in Fig. 1. In particular, the process was divided into four steps: data acquisition (1), data collection and anonymization (2), data labeling and annotation (3) and data evaluation and extraction (4).

### Data acquisition

The data acquisition was performed by five radiologists/oncologists working at medical centers in Poland: the Breast Unit, Lower Silesian Oncology, Pulmonology and Hematology Center and Maria Sklodowska-Curie National Institute of Oncology - National Research Institute, Branch in Krakow. For image acquisition, the following ultrasound scanners were used:

- Hitachi ARIETTA 70 equipped with linear array transducer L441 (frequency range: 2–12 MHz);
- Esaote 6150 equipped with linear array transducer LA523 (frequency range: 4–13 MHz);
- Samsung RS85 equipped with linear array transducer L3–12A (frequency range: 3–12 MHz);
- Philips Affiniti 70 G and EPIQ 5 G equipped with linear array transducers eL18-4 (frequency range: 2–22 MHz) and L12-5 (frequency range: 5–12 MHz).

The breast ultrasound examination was conducted in accordance with the BI-RADS guidelines of the ACR. The ultrasound device settings (e.g. transmit frequency or gain) were individually chosen for the patient to obtain a tumor image appropriate for interpretation. In case of suspicion of malignancy, a core needle biopsy was performed.

### Data collection and anonymization

In building the dataset, the first clinically non-standard step was to anonymize, collect and transfer the data. To protect patients’ privacy, all identifiable information has been removed from the images.

The anonymization was conducted at the institutions of the data origin. For each file, all DICOM tags containing sensitive or identifiable information such as patient ID, patient’s name, or patient’s date of birth were deleted or replaced with random values. Then, all patient-related textual information within the image (e.g. patient ID) was permanently removed. Before transferring, all anonymized images were manually reviewed to ensure that all information had been removed.

Of the DICOM tags, only those image-related (i.e. Width, Height, Bit Depth, Samples Per Pixel, Photometric Interpretation, Bits Allocated, Bits Stored, High Bit, Pixel Representation, Derivation Description, Pixel Data and Sequence Of Ultrasound Regions - Region Location Min X0, Region Location Min Y0, Region Location Max X1, Region Location Max Y1, Physical Units X Direction, Physical Units Y Direction, Physical Delta X, Physical Delta Y) were preserved, as they are necessary for the proper displaying of the image and its subsequent analysis. To facilitate this workflow, we designed and created a purpose-built web-based platform for collecting, annotating, and labeling breast ultrasound images.

### Data labels and annotation

During data acquisition, patient clinical data were collected, such as age, breast tissue composition, signs, and symptoms. They were paired with the image during data uploading. The list of labels for signs/symptoms consisted of the most prevalent observed abnormalities/reported experiences. Labels of tissue composition are in accordance with BI-RADS guidelines^3^.

**Fig. 2:**
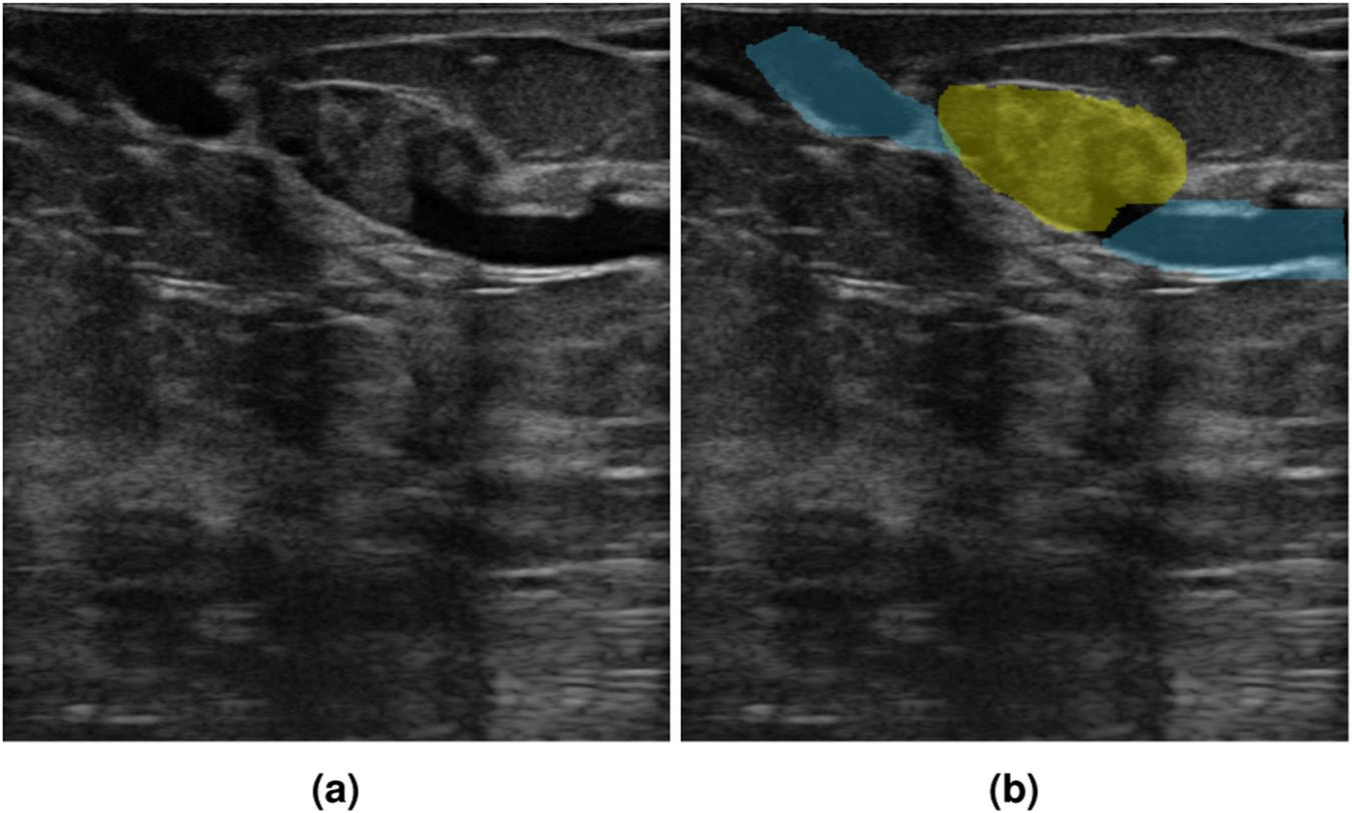
An example of a breast lesion image (**a**) and its segmentation into two classes (**b**), tumor area (marked in yellow) and areas of other abnormalities (marked in blue).

Next, the radiologist, who collected the data, indicated the regions of interest using freehand annotations. In segmentation, two tissue classes were considered: (1) the tumor mask which outlined the mass under examination, and (2) the other object mask which was optional and concerned other suspicious lesions in the image (e.g. cyst). The boundary of the segmented lesion is in line with the measurement markers of the lesion size used during standard ultrasound scanning. For normal cases, the masks are not available due to the lack of abnormal findings. The example of image segmentation with two classes of masks is shown in Fig. 2, yellow represents the tumor class, and blue – the other object class. For annotation, each radiologist chose a tablet with a pencil or a computer with a mouse depending on their preference.

Image annotating was followed by labeling according to BI-RADS reporting guidelines^3^. Seven B-mode-based features were included. They were divided into mass-oriented features (shape, margin, echogenicity, posterior features, halo) and image-oriented features (calcifications, skin thickening). Labels of tumor orientation (parallel/not parallel) were excluded because their direct interpretation is provided in the tumor mask. For normal cases, six features are not applicable, so only skin thickening is considered. This BI-RADS reporting section was ended by assigning one of seven categories (BI-RADS 1, 2, 3, 4a, 4b, 4c, 5). In addition, each tumor was labeled for 15 image interpretations that reflect the radiologist’s overall diagnostic impression. This list of interpretations consisted of the most prevalent diseases that are differentiated in clinical practice.

The last group of labels is associated with the method of tumor verification (follow-up care/biopsy), histologic diagnosis, and final classification (benign/malignant). The list of labels for histologic type of tumor was prepared in accordance with the ICD-10^22^ and the 5th edition WHO Classification of Breast Tumors^23^.

### Data evaluation and extraction

Finally, all collected data were evaluated in terms of preparation for export. Inclusion criteria were defined as female patients with tumor type confirmed by pathological diagnosis or over 2-year follow-up care. Moreover, the final dataset includes only B-mode breast images with tumors not exceeding image size, without measurement markers, pictograms, artifacts, and text annotations.

All images have been cropped to remove text annotations with device settings on the image sides. In non-rectangular images (from extended field-of-view imaging), the black background in the frame has been changed to transparent (see the image of BIRADS 2 in Table 2) to allow analyses requiring data limited to the image itself. Ignoring the alpha channel, the background remains black.

**Table 2:** Examples of images with overlaid annotations indicating lesions areas for each BI-RADS category.

BIRADS	Image	Image with overlaid annotation - semitransparent yellow tumor mask (if applicable)
1		
2		
3		
4a		
4b		
4c		
5		

Each of the five radiologists contributed equally to the final dataset. The final extraction of the dataset is de-identified in radiologist ID terms, the case is no longer associated with the radiologist. As a result, the radiologist-medical center-patient linkage is removed, so the patients’ identities cannot be reasonably determined from the provided data.

## Data Records

The BrEaST dataset has been made available for download at The Cancer Imaging Archive (TCIA)^21^ and for viewing on the dedicated webpage^24^. Additionally, thumbnail preview of all images and lesion masks is included in supplementary material attached to this paper.

### Data characteristics

**Fig. 3:**
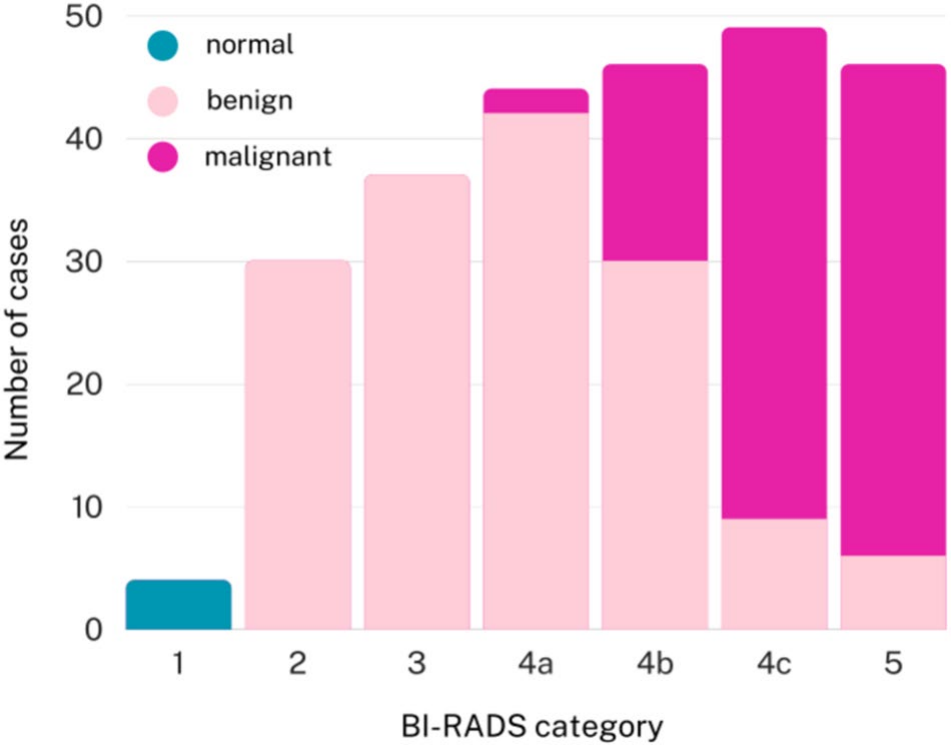
Distribution of normal, benign and malignant cases for all BI-RADS categories.

The data were acquired from 256 adult female patients between 18 and 87 years old at the examination time. A total of 197 biopsies were performed (accounting for 77% of the dataset), confirming 98 breast cancers. The biopsy results available for BIRADS 3 (12 such cases) are diagnoses made prior to follow-up ultrasound scanning (during which the images were acquired). Conclusively, the dataset consists of 98 cancers, 154 benign lesions and 4 normal tissue images. The number of images of these classes for all BIRADS categories is shown in Fig. 3.

Examples of images from each BIRADS category with overlying annotations defining the tumor area (if applicable) are shown in Table 2. The selected images show the diversity of the released dataset. The image of BIRADS 1 shows normal breast tissue during lactation with clearly widened milk ducts. The BIRADS 2 image shows the lymphocele in the post-breast-conserving therapy setting. The image was acquired in extended field-of-view mode, so transparency is added to its sides. The image of BIRADS 3 shows the tumor above the silicone implant. The images of BIRADS 4a, 4b, 4c,and 5 show lobular carcinoma *in situ*, mastitis, invasive carcinoma of no special type, and invasive carcinoma of no special type with foci of sebaceous carcinoma, respectively.

The data characteristics, including all considered attributes with their definition and the prevalence of each label, are summarized in Tables 3, 4. It should be emphasized that the Diagnosis column (of Table 4) contains values “not applicable” due to the absence of a histopathological result for cases of BIRADS categories 1,2,3 (for BIRADS 4a, 4b, 4c, 5, the column is completely filled in). In the absence of a histopathological diagnosis, the Interpretation column showing the radiologist’s overall diagnostic impression should be used (also completely filled in for BIRADS 2–5, not applicable for BIRADS 1).

**Table 3:** The dataset characteristics for clinical patient attributes and BI-RADS descriptors.

Attribute	Description	Range of values (number of cases)	Multiple selection
CaseID	unique identifier	1–256	no
Age	patient age	• 18–87 (mean: 53; median: 55; standard deviation: 16) (n = 214)	no
		• not available (n = 42)	
Tissue composition	breast tissue composition assessed during examination	• heterogeneous: predominantly fat (n = 80)	yes
		• heterogeneous: predominantly fibroglandular (n = 56)	
		• homogeneous: fibroglandular (n = 46)	
		• homogeneous: fat (n = 24)	
		• lactating (n = 7)	
		• not available (n = 49)	
Signs	objective observable abnormalities perceived by physician	• no (n = 147)	yes
		• palpable (n = 58)	
		• breast scar (n = 5)	
		• skin retraction (n = 4)	
		• warmth (n = 4)	
		• nipple retraction (n = 3)	
		• redness (n = 3)	
		• peau d’orange (n = 1)	
		• not available (n = 46)	
Symptoms	subjective experiences reported by patient	• no (n = 113)	yes
		• family history of breast/ovarian cancer (n = 22)	
		• HRT/hormonal contraception (n = 17)	
		• nipple discharge (n = 7)	
		• personal history of breast cancer (n = 3)	
		• breast injury (n = 1)	
		• not available (n = 98)	
Shape	BI-RADS descriptor	• irregular (n = 140)	no
		• oval (n = 97)	
		• round (n = 15)	
		• not applicable (n = 4)	
Margin	BI-RADS descriptor	• circumscribed (n = 115)	yes
		• not circumscribed - indistinct (n = 115)	
		• not circumscribed - angular (n = 42)	
		• not circumscribed - microlobulated (n = 36)	
		• not circumscribed - spiculated (n = 33)	
		• not applicable (n = 4)	
Echogenicity	BI-RADS descriptor	• hypoechoic (n = 148)	no
		• heterogeneous (n = 57)	
		• anechoic (n = 15)	
		• isoechoic (n = 12)	
		• complex cystic/solid (n = 11)	
		• hyperechoic (n = 9)	
		• not applicable (n = 4)	
Posterior features	BI-RADS descriptor	• no (n = 159)	no
		• shadowing (n = 50)	
		• enhancement (n = 36)	
		• combined (n = 7)	
		• not applicable (n = 4)	
Halo	BI-RADS descriptor	• no (n = 197)	no
		• yes (n = 55)	
		• not applicable (n = 4)	
Calcifications	BI-RADS descriptor	• no (n = 225)	no
		• in a mass (n = 23)	
		• intraductal (n = 2)	
		• indefinable (n = 2)	
		• not applicable (n = 4)	
Skin_thickening	BI-RADS descriptor	• no (n = 241)	no
		• yes (n = 15)

**Table 4:** The dataset characteristics (contd.) for final image attributes and histologic diagnosis.

Attribute	Description	Distinct collection of values (number of cases)	Multiple selection
Interpretation	image interpretation for mass differentiation	• Suspicion of malignancy (n = 149)	yes
		• Fibroadenoma (n = 101)	
		• Intraductal papilloma (n = 68)	
		• Dysplasia (n = 66)	
		• Cyst filled with thick fluid (n = 31)	
		• Complex cyst (n = 27)	
		• Mammary duct ectasia (n = 13)	
		• Duct filled with thick fluid (n = 12)	
		• Hamartoma (n = 8)	
		• Breast scar (surgery) (n = 6)	
		• Mastitis (n = 6)	
		• Hematoma (n = 4)	
		• Intramammary lymph node (n = 4)	
		• Lipoma (n = 4)	
		• Silicone implant (n = 4)	
		• Simple cyst (n = 4)	
		• Adenosis (n = 2)	
		• Fat necrosis (n = 2)	
		• Implant rupture (n = 2)	
		• Lacteal cyst (n = 2)	
		• Phyllodes tumor (n = 2)	
		• Abscess (n = 1)	
		• Breast scar (radiotherapy) (n = 1)	
		• Hemangioma (n = 1)	
		• Isolated calcifications (n = 1)	
		• Lactating adenoma (n = 1)	
		• Seroma (n = 1)	
		• not applicable (n = 4)	
BIRADS	BI-RADS category	• 1 (n = 4)	no
		• 2 (n = 30)	
		• 3 (n = 37)	
		• 4a (n = 44)	
		• 4b (n = 46)	
		• 4c (n = 49)	
		• 5 (n = 46)	
Verification	confirmation method for mass classification	• confirmed by biopsy (n = 197)	no
		• confirmed by follow-up care (n = 55)	
		• not applicable (n = 4)	
Diagnosis	histological type of mass	• Invasive carcinoma of no special type (NST) (n = 66)	yes
		• Fibroadenoma (n = 30)	
		• Benign mammary dysplasia (n = 27)	
		• Ductal carcinoma *in situ* (DCIS) (n = 14)	
		• Invasive lobular carcinoma (n = 13)	
		• Fibrosclerosis (n = 11)	
		• Intraductal papilloma (n = 8)	
		• Usual ductal hyperplasia (UDH) (n = 7)	
		• Pseudoangiomatous stromal hyperplasia (PASH) (n = 6)	
		• Invasive micropapillary carcinoma (n = 4)	
		• Mucinous carcinoma (n = 4)	
		• Cribriform carcinoma (n = 3)	
		• Phyllodes tumor (n = 3)	
		• Encapsulated papillary carcinoma (n = 2)	
		• Fibrocystic change (n = 2)	
		• Lobular carcinoma *in situ* (LCIS) (n = 2)	
		• Mastitis (n = 2)	
		• Tubular carcinoma (n = 2)	
		• Adenosis (n = 1)	
		• Apocrine carcinoma (n = 1)	
		• Atypical lobular hyperplasia (ALH) (n = 1)	
		• Complex sclerosing lesion (n = 1)	
		• Fat necrosis (n = 1)	
		• Fibroadenosis (n = 1)	
		• Hamartoma (n = 1)	
		• Intramammary lymph node (n = 1)	
		• Invasive papillary carcinoma (n = 1)	
		• Lactating adenoma (n = 1)	
		• Lymphoma (n = 1)	
		• Metaplastic carcinoma (n = 1)	
		• Sebaceous carcinoma (n = 1)	
		• Simple cyst (n = 1)	
		• Solid papillary carcinoma *in situ* (n = 1)	
		• not applicable (n = 59)	

### Dataset structure

The downloaded files are (1) a *.zip* file containing a folder with images and masks, and separately (2) a*.xlsx* file with labels.

- The folder comprises all images and their corresponding segmentations of tumors (files ending with _tumor.png) and segmentations of other areas (files ending with _other.png)
- The file*.xlsx* contains 257 rows (the first row with column headers and 256 rows with cases data). The rows of the*.xlsx* file represent consecutive cases with the following attributes: case identifier (Case_ID), the filename of the image (Image_filename), the filename of tumor annotation (Mask_tumor_filename), the filenames of other objects annotations (Mask_other_filename), the width and height of pixel in cm (Pixel_size), patient age (Age), type of breast tissue composition (Tissue_composition), observed signs (Signs), reported symptoms (Symptoms), tumor shape (Shape), tumor margin (Margin), tumor echogenicity (Echogenicity), posterior features (Posterior_features), presence of hyperechoic halo (Halo), presence of calcifications (Calcifications), presence of skin thickening (Skin_thickening), radiologist interpretation (Interpretation), BI-RADS category (BIRADS), method of tumor verification (Verification), histologic diagnosis (Diagnosis), final tumor classification (Classification).

The examples of rows with data descriptions are presented in Table 5. For multiple-choice attributes, the selected labels are concatenated using the ‘&’ character (see Symptoms or Diagnosis column in Table 5). A slightly different notation is used for multiple choices in the margin field. Since ‘not circumscribed’ includes the labels of subcategories, the notation method has been changed to concatenate the labels using ‘&’ and adding ‘not circumscribed’ before the concatenated phrase (e.g., ‘not circumscribed - angular&indistinct’, see Margin column in Table 5). For images with multiple annotations, the filenames are also concatenated using the ‘&’ character, whereas no mask is provided, the field is left blank (see Mask_other_filename column in Table 5).

**Table 5:** Examples of two rows (for better readability shown as columns) from the .csv file describing the dataset.

No.	CSV file column name	Example #1	Example #2
1	Case_ID	151	225
18	Image_filename	case151.png	case225.png
19	Mask_tumor_filename	case151_tumor.png	case225_tumor.png
20	Mask_other_filename	case151_other1.png**&**case151_other2.png	
21	Pixel_size	0.0069444444961845875	0.0078125
2	Age	43	63
3	Tissue_composition	heterogeneous, predominantly fibroglandular	heterogeneous, predominantly fibroglandular
4	Signs	no	palpable
5	Symptoms	nipple discharge**&**family history of breast/ovarian cancer	HRT/hormonal contraception
6	Shape	oval	irregular
7	Margin	circumscribed	not circumscribed - angular**&**indistinct
8	Echogenicity	hyperechoic	heterogeneous
9	Posterior_features	no	no
10	Halo	no	yes
11	Calcifications	no	no
12	Skin_thickening	no	no
13	Interpretation	Duct filled with thick fluid**&**Mammary duct ectasia**&**Intraductal papilloma	Suspicion of malignancy
14	BIRADS	4a	5
15	Verification	confirmed by biopsy	confirmed by biopsy
16	Diagnosis	Intraductal papilloma	Invasive carcinoma of no special type (NST)**&**Apocrine carcinoma
17	Classification	benign	malignant

The ‘&’ sign separates multiple elements in a field.

## Technical Validation

The quality of the BrEaST dataset^21^ was prompted by controlling each stage of data processing and analysis. The validation process was divided into three parts: (1) regular validation of the dataset performed during the dataset development, (2) validation of annotations to check their usability in analyses, (3) simple analysis to validate the association of annotations with labels.

### Regular validation

The web-based purpose-built system had the fully controlled workflow and allowed radiologists to validate each stage of data processing and to report errors on an ongoing basis (e.g. improper anonymization of data). Furthermore, error handling was implemented as part of the annotation and validation framework to prevent mechanical errors (e.g., skipping the BI-RADS category when tumor annotations were completed). Finally, submitting the form with labels and annotations required double confirmation to deter accidental clicks and ensure that blank fields were unavailable information and not omissions.

After the dataset was collected and fully described, it was manually checked by a database manager and then cross-checked by another expert. The final step was checking the dataset for duplicates, using a previously developed algorithm^20^.

### Validation of tumor annotations

**Fig. 4:**
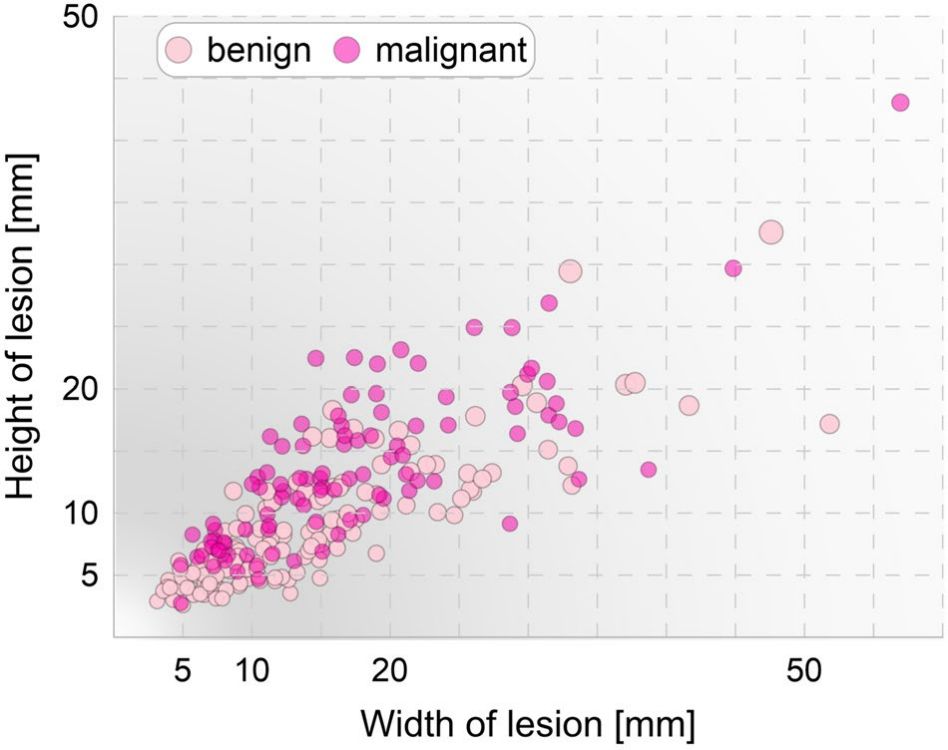
The height and width of the tumors determined from masks drawn by radiologists. The grayscale background shows the probability density function computed from the maximum diameter.

First, the process of annotation validation included checking whether each mask consisted of a single object. Binarization of the drawn contours sometimes set random pixels as belonging to an object (e.g. resulting from resting a hand on a tablet screen). Then, the height and width of the tumor were automatically determined based on the masks according to the ACR guidelines^3^. The derived measurements (and corresponding masks) were verified by each radiologist. For all the masks discussed, the original ones were kept as the radiologist who performed the examination had the greatest knowledge of the lesion in question. The resulting 252 pairs of measurements are presented in Fig. 4.

The results obtained are in line with expectations. Markers of malignant tumors are clearly clustered over those of benign ones. This observation is reflected in the BI-RADS feature, i.e. in orientation, where non-parallel property (vertical dimension is greater than horizontal dimension) is a predictor of malignancy. The longest diameter was also taken into account (the distribution of its values is shown in grayscale background in Fig. 4), as it is a clinically used measurement in assessment of the tumor response to treatment (also used in ultrasound^25^) and is also evaluated in Tumor Node Metastasis (TNM) staging^26^.

Moreover, the higher density of points can be seen at small tumor sizes. Considering the thresholds from the TNM staging, the size distribution is as follows 10 cases within T1a stage, 76 within T1b, 107 within T1c, 57 within T2, and 2 within T3. Therefore, 77% (n = 193) of the determined tumor dimensions is classified as T1. The skewness (equals to 1.44) of the dataset toward smaller tumors enables the development of methods to detect them at an earlier stage. Additionally, earlier diagnosis of carcinomas is crucial for effective treatment.

### Validation of annotations and labels

An example of quantitative analysis is the assessment of tumor shape based on the masks included in the collection. One of the primary methods for evaluating shape roughness and complexity is the turning angle function (TAF)^27^ of a contour, which simplifies the characterization of shapes and can be used as their signature. It is the cumulative function of turning angles, and it may be obtained by deriving the counterclockwise angle between the tangent at the segment of a contour and the x-axis, and expressing it as a function of the arc length of the segment. The perimeter of the lesions presented in Table 6 was smoothed by using a moving average based on 10 points for a clearer presentation of the TAF.

**Table 6:** Examples of images with overlaid annotations indicating smoothed lesions’ perimeter and turning angle change as modified Turning Angle Function (last column).

BIRADS (shape/margin)	Image with lesion perimeter	Turning Angle Function
2 (oval/circumscribed)		
3 (oval/circumscribed)		
4a (oval/circumscribed)		
4b (irregular/circumscribed)		
4c (irregular/not circumscribed - angular & indistinct)		
5 (irregular/not circumscribed - angular & indistinct)		

## Usage Notes

The BrEaST dataset is available for download at TCIA^21^ and for browsing (as an atlas of breast lesions along with histological diagnoses) on the dedicated webpage^24^. It was created to develop and evaluate algorithms for detecting, segmenting, and classifying abnormalities in breast ultrasound scans. Applications of the BrEaST dataset may include:

- Training and testing models for localizing lesions in images (available masks for multi-tumor images, non-cropped images with skin layer and no visible markers);
- Training and testing models for segmenting lesions in images (provided masks created manually by experienced radiologists);
- Training and testing models for classifying lesions in images (available BI-RADS category and classification into benign/malignant);
- Testing methods using the dataset as a benchmark what can increase the interpretability of the models’ performance by filtering or grouping labels (available e.g. BIRADS features, diagnoses, interpretations, signs and symptoms).

The released dataset has some limitations that need to be addressed in the future, including:

- The number of cases for some diagnoses is limited due to their rare prevalence in the population (e.g., invasive papillary carcinoma or sebaceous carcinoma). Therefore, training machine learning algorithms on the BrEaST dataset to diagnose rare diseases may be unbalanced. Albeit it is useful information that, added to the benign/malignant labels, expands the field of research. For example, it enables grouping of tumors by invasiveness (non-invasive vs. pre-invasive vs. invasive lesions) to enhance the interpretability of the lesion classification model (e.g., misclassification of pre-invasive lesions).
- Only few normal cases (no lesion present) are included in the database for models evaluation, but these cases can be supplemented from other sources (Table 1).

## Data Availability

The custom code for importing dataset into variables in Matlab environment (Mathworks, USA) and Python programming language is available at github repository^28^. The code used for processing DICOM images was based on the cornerstone3D^29^, dicomParser^30^ and Nanodicom^31^ libraries. The code used for image annotation was based on markerjs2^32^ library.
